# Real-world use of avatrombopag in patients with chronic liver disease and thrombocytopenia undergoing a procedure

**DOI:** 10.1097/MD.0000000000035208

**Published:** 2023-10-06

**Authors:** Sanjaya K. Satapathy, Vinay Sundaram, Mitchell L. Shiffman, Brian D. Jamieson

**Affiliations:** a North Shore University Hospital, Northwell Health, Manhasset, NY; b Donald and Barbara Zucker School of Medicine at Hofstra/Northwell Health, Hempstead, NY; c Division of Gastroenterology and Comprehensive Transplant Center, Cedars-Sinai Medical Center, Los Angeles, CA; d Liver Institute of Virginia, Liver Institute of Richmond, Liver Institute of Hampton Roads, Bon Secours Mercy Health, Richmond and Newport News, VA; e Sobi™, Inc., Durham, NC.

**Keywords:** cirrhosis, periprocedural, platelet, thrombopoietin receptor agonist, transfusion

## Abstract

The phase 4 observational cohort study assessed the effectiveness and safety of the thrombopoietin receptor agonist avatrombopag in patients with chronic liver disease (CLD) and thrombocytopenia undergoing a procedure. Patients with CLD may have thrombocytopenia, increasing the risk of periprocedural bleeding. Prophylactic platelet transfusions used to reduce this risk have limitations including lack of efficacy and transfusion-associated reactions. Prophylactic thrombopoietin receptor agonists have been shown to increase platelet counts and decrease platelet transfusions. Effectiveness was assessed by change from baseline in platelet count and proportion of patients needing a platelet transfusion. Safety was assessed by monitoring adverse events (AEs). Of 50 patients enrolled, 48 were unique patients and 2 patients were enrolled twice for separate procedures. The mean (standard deviation) change in platelet count from baseline to procedure day was 41.1 × 10^9^/L (33.29 × 10^9^/L, n = 38), returning to near baseline at the post-procedure visit (change from baseline −1.9 × 10^9^/L [15.03 × 10^9^/L], n = 11). The proportion of patients not requiring a platelet transfusion after baseline and up to 7 days following the procedure was 98% (n = 49). Serious AEs were infrequent (n = 2 [4%]). No treatment-emergent AEs were considered related to avatrombopag. There were 2 mild bleeding events, no thromboembolic events or deaths, and no patients received rescue procedures (excluding transfusions). This study found that in a real-world setting, treatment with avatrombopag was well tolerated, increased the mean platelet count by procedure day, and reduced the need for intraoperative platelet transfusions in patients with CLD and thrombocytopenia.

## 1. Introduction

Chronic liver disease (CLD) is associated with hematological abnormalities,^[1]^ the most common being thrombocytopenia,^[2,3]^ defined as a platelet count < 150 × 10^9^/L, with severe thrombocytopenia defined as a platelet count < 50 × 10^9^/L.^[2]^ The main causes of thrombocytopenia in patients with CLD are splenic sequestration and the decreased production of thrombopoietin by the liver.^[2]^ Reduced hepatic thrombopoietin synthesis in patients with CLD results in a reduction of megakaryocytopoiesis, thrombopoiesis, and platelet release into the circulation.^[2]^

Thrombocytopenia in patients with CLD is nearly always linked to cirrhosis,^[2]^ which is the common final stage in CLD progression, resulting in platelet counts decreasing as the severity of cirrhosis increases.^[4]^ There are an estimated 4 million adults with CLD in the USA (1.6% of the population).^[5]^ Moreover, the incidence of thrombocytopenia in patients with CLD without cirrhosis is 6%,^[5]^ whereas in patients with cirrhosis it can be as high as 92%.^[3]^

Invasive surgical and therapeutic procedures, such as liver biopsies, variceal band ligation, or percutaneous procedures for hepatocellular carcinoma are common in patients with CLD.^[6]^ However, thrombocytopenia increases the risk of periprocedural bleeding,^[6,7]^ which can result in hospitalizations, disability, and absenteeism.^[8]^ Peer-reviewed literature and expert guidance on the management of thrombocytopenia in patients with CLD is limited^[5]^ and historically treatment options were limited to platelet transfusion.^[9]^

Prophylactic platelet transfusion is commonly used to improve thrombocytopenia in patients with CLD,^[2,10]^ but this approach is limited as the response decreases with each subsequent transfusion.^[11]^ Platelet transfusions also expose patients to risks of transfusion reactions and infections,^[2,5,9]^ development of antiplatelet antibodies,^[2,5,9]^ and increased portal hypertension.^[5]^

Given the drawbacks of traditional treatments, focus has shifted to the use of thrombopoietin receptor agonists (TPO-RAs), which interact with the thrombopoietin receptor on megakaryocytes resulting in an increase in platelet production.^[5]^ Initially TPO-RAs were developed to increase platelet counts in patients with immune thrombocytopenia^[12,13]^ and more recently 2 TPO-RAs, avatrombopag and lusutrombopag, were approved by the US Food and Drug Administration (FDA)^[14,15]^ and European Medicines Agency^[16,17]^ for the prophylactic treatment of patients with CLD-associated thrombocytopenia who are undergoing a procedure.^[18,19]^

Recent meta-analyses indicated that the use of TPO-RAs prior to procedures results in increased platelet counts and decreased incidence of platelet transfusions, compared with placebo, whilst having no significant effect on the rate of portal vein thrombosis.^[18,20]^ Avatrombopag is an oral TPO-RA that is indicated for the treatment of thrombocytopenia in patients with CLD prior to a scheduled procedure.^[14]^ In 2 phase 3 randomized, placebo-controlled trials, ADAPT-1 and ADAPT-2, avatrombopag was shown to be superior to placebo in reducing the need for platelet transfusions and rescue procedures for bleeding^[21]^ and was well tolerated, with a generally comparable safety profile to that of placebo.^[22]^

This phase 4 observational cohort study was designed to assess the real-world effectiveness, safety, and treatment patterns of avatrombopag in patients with thrombocytopenia associated with CLD undergoing a procedure.

## 2. Materials and methods

### 2.1. Study design

This was a phase 4, multicenter, observational cohort study (NCT03554759) conducted from July 2018 to January 2019. The study planned to enroll a total of 500 subjects from sites in the USA after avatrombopag was approved for this indication by the FDA in May 2018.^[14]^ All treatment decisions were at the discretion of the treating physician as per routine medical care and were not mandated by study design or protocol. The protocol, informed consent form and any appropriate related documents were submitted to the Institutional Review Boards (IRB; the 2 central IRBs were Copernicus Group IRB and Western IRB, with 12 local IRBs utilized at the local level) by the study principal investigators for review and approval, and the study was initiated after the principal investigators and the Sponsor received approval of the protocol and the informed consent form.

Data were collected prospectively or retrospectively from information routinely recorded in a patient’s medical records and from laboratory data. Visits, examinations, laboratory tests, or procedures were not mandated or recommended as part of this study. The duration of patient participation and collection of clinical data was up to 6 weeks from the initial (baseline) visit or data were extracted from an approximately 6-week window of patient visits. Data were entered into the electronic data capture system based on patient visits occurring within approximately 7 calendar days of their first avatrombopag use (baseline visit), during any visits while taking avatrombopag (treatment period), on procedure day, on discharge day (if applicable) and for any visit performed up to 30 days post-procedure (follow-up period).

### 2.2. Inclusion criteria

All patients enrolled were ≥ 18 years, had thrombocytopenia associated with CLD and were planned to undergo, or underwent, treatment with avatrombopag prior to a procedure. For retrospective enrollment (patients enrolled and consented after procedure day) patients must have had, at a minimum, a platelet count from approximately 7 days prior to starting avatrombopag and a platelet count on the procedure day, to enable evaluation of study endpoints. All patients provided written informed consent and there were no exclusion criteria for participation in this observational study, including no exclusion of patients for concomitant medications before or during the study.

### 2.3. Treatment

Avatrombopag was taken orally and doses were determined by the treating physician in conjunction with the FDA-approved US prescribing information.^[14]^ The recommended dosing of avatrombopag is for 5 consecutive days starting 10 to 13 days prior to a scheduled procedure (with the procedure occurring within 5 to 8 days after the last dose), at a dose of 40 mg for patients with a platelet count between ≥ 40 × 10^9^/L and < 50 × 10^9^/L and 60 mg for patients with a platelet count < 40 × 10^9^/L. Avatrombopag was not provided by the sponsor; participating patients received the commercially available drug through a prescription written by a healthcare provider as per standard of care.

### 2.4. Effectiveness analysis

The effectiveness of avatrombopag was assessed by the change from baseline in platelet count on procedure day and the proportion of patients who received a platelet transfusion after the baseline visit and up to 7 days post-procedure day. The effectiveness of avatrombopag was also assessed by subgroup analyses of the change in platelet count from baseline to procedure day by baseline platelet count group and Child–Turcotte–Pugh (CTP) Grade.

Additional ad hoc analyses, not prespecified in the final statistical analysis plan, were also performed. These included a responder analysis based on patients achieving platelet count ≥ 50 × 10^9^/L on procedure day, divided into 2 groups of patients with a baseline platelet count of < 40 × 10^9^/L or patients with a baseline count of ≥ 40 to < 50 × 10^9^/L. The same responder analysis was performed on the subset of patients receiving correct dosing of avatrombopag as per the US prescribing information (excluding patients that received off-label avatrombopag).

### 2.5. Safety analysis

The safety of avatrombopag was assessed by recording adverse events (AEs). These were reported by the patient or, when appropriate, by a caregiver, surrogate, or the patient’s legally authorized representative, and/or collected from data recorded in the patient’s medical record. The severity of each AE was recorded, with mild AEs defined as transient, requiring minimal treatment or intervention and not interfering with daily living; moderate AEs defined as being alleviated with specific therapeutic intervention causing some impairment to daily activities and discomfort but posing no significant or permanent risk of harm; and serious AEs defined as AEs resulting in death, a threat to life, hospitalization (even if admitted and discharged on the same day, although an emergency room attendance that did not result in admission was not included), or prolongation of existing hospitalization, a persistent or significant disability, a congenital anomaly, or a requirement for medical or surgical intervention to prevent any of the aforementioned criteria.

AEs were deemed to be treatment-emergent AEs (TEAEs) or serious TEAEs when the time course between the administration of avatrombopag and the occurrence or worsening of the AE was consistent with a causal relationship and no other cause (concomitant drugs, therapies, complications, etc) could be identified. The AEs of special interest were defined as thromboembolic events (any thrombotic or embolic event, whether arterial or venous) and bleeding events (any clinically significant blood loss).

### 2.6. Statistical analysis

The sample size for this study was based on clinical, rather than statistical rationale, and was considered adequate to address the study objective, which was to observe the treatment patterns and effects of avatrombopag in real-world practice. This objective was neither related to the testing of a specific hypothesis, nor to the precision of a particular estimate. The analysis population and the safety population analyzed were defined as all enrolled patients. The analysis of effectiveness endpoints was descriptive and based on data entered in the electronic case report form for enrolled patients. A 95% exact confidence interval (CI) (using the Clopper–Pearson method) was performed for the proportion of patients who received a platelet transfusion after the baseline visit and up to 7 calendar days following procedure day.

## 3. Results

### 3.1. Patient population

This phase 4 observational registry study was conducted at 43 sites in the USA and was terminated by the sponsor, due to enrollment challenges, prior to completing the planned enrollments. When terminated, 29 of the 43 active sites had screened a total of 65 patients, and 25 of the sites had enrolled a total of 50 patients. Of these 50 patients, 48 were unique patients with 2 patients having been reenrolled into the study and received 2 regimens of avatrombopag for separate procedures, as allowed by the protocol.

The overall mean (standard deviation [SD]) age was 61.2 (8.28) years with a higher proportion of males (56%) than females. The enrolled patients had a mean (SD) CTP Grade of 6.7 (1.55) (n = 38) and were of predominantly white racial heritage; full demographics and baseline characteristics are shown in Table 1. The mean (SD) baseline platelet count was 46.9 × 10^9^/L (24.52 × 10^9^/L). A total of 20 (40%) patients had a baseline platelet count < 40 × 10^9^/L and 42% of patients (n = 21) had a baseline platelet count between 40 × 10^9^/L and < 50 × 10^9^/L. A total of 9 (18%) patients had a baseline platelet count ≥ 50 × 10^9^/L, and of these 4 (8%) had a baseline platelet count ≥ 100 × 10^9^/L. The most common etiologies of liver disease were chronic hepatitis C virus (n = 17; 34%), alcoholic liver disease (n = 13; 26%), nonalcoholic steatohepatitis (n = 11; 22%), and chronic hepatitis B virus (n = 2; 4%). A total of 40 patients had a reported CTP Grade at baseline, of which 26 (65%) had a CTP Grade of A, 11 (27.5%) had a CTP Grade of B, and 3 (7.5%) had a CTP Grade of C.

**Table 1:** Demographics and baseline characteristics.

Demographics and baseline characteristics	N = 50
Age, yr, median (range)	61.5 (44–80)
Sex, n (%)	
Male	28 (56)
Female	22 (44)
Race, n (%)	
White	41 (82)
Black or African American	4 (8)
Asian	3 (6)
Other	2 (4)
Baseline platelet count, ×10^9^/L, median (range)	43 (14–137)
Mean (SD)	47 (25)
Baseline platelet count subgroup, ×10^9^/L, n (%)	
<20	2 (4)
20–<30	5 (10)
30–<40	13 (26)
40–<50	21 (42)
50–<100	5 (10)
≥100	4 (8)
Any previous platelet transfusions, n (%)	13 (26)
Any previous avatrombopag use, n (%)	5 (10)
Etiology of liver disease,* n (%)	
Alcoholic liver disease	13 (26)
Chronic hepatitis B	2 (4)
Chronic hepatitis C	17 (34)
Non-alcoholic steatohepatitis	11 (22)
Other†	14 (28)
Child–Turcotte–Pugh Grade, n (%)	
A (5–6 points)	26 (52)
B (7–9 points)	11 (22)
C (10–15 points)	3 (6)
Missing	10 (20)
Model for end-stage liver disease score (n = 45), median (range)	12 (6-26)
Patient has hepatocellular carcinoma, n (%)	5 (10)‡
Patients with invasive procedures in the previous 12 mo, n (%)	22 (44)
Number of invasive procedures, median (range)	1 (1–9)

Percentages are based on the number of enrolled patients.

SD = standard deviation.

* Patients may be counted in more than one category.

† Other etiology of CLD included cryptogenic cirrhosis (n = 4), autoimmune hepatitis (n = 3), hepatic cirrhosis (n = 2), primary sclerosing cholangitis (n = 1), biliary cirrhosis (n = 1), primary biliary cholangitis (n = 1), hepatoportal sclerosis (n = 1) and cirrhosis of the liver (n = 1).

‡ Barcelona clinic liver cancer Grade for hepatocellular carcinoma included: Grade 0 (n = 1), Grade A (n = 2), Grade B (n = 1) and unknown (n = 1).

All patients completed a 5-day course of once-daily avatrombopag, with 1 (2%) receiving 20 mg (off-label), 27 (54%) receiving 40 mg, and 22 (44%) receiving 60 mg. All patients received at least 1 concomitant medication during the study, which included medications administered during the procedure (41 [82%] receiving a concomitant medication on procedure day) and for the treatment of AEs. Most concomitant medications were within the pharmacological subclasses of anesthetics and drugs for acid-related disorders.

### 3.2. Types of procedures

Procedures that occurred during the study could be defined as either the primary procedure or the secondary procedure when multiple procedures occurred at the same time. The most common procedure was upper gastrointestinal (GI) endoscopy (56% [n = 28] of primary procedures and 4% [n = 2] of secondary procedures). Of the 7 procedures classified as “other,” 2 were right inguinal hernia repairs, 1 cervical epidural injection, 1 right L3 to L4 microdiscectomy and 1 endometrial curette (all primary), and 1 umbilical hernia repair and 1 sigmoidoscopy (secondary) (Table 2). A total of 2 patients were enrolled twice for separate procedures, with 1 patient undergoing a GI endoscopy with variceal sclerotherapy followed approximately 6 weeks later by a GI endoscopy without biopsy, and the other patient undergoing a GI endoscopy with variceal banding that was repeated approximately 8 weeks later. No patient had a delayed discharge due to postoperative bleeding or thrombocytopenia.

**Table 2:** Surgical procedures.

Surgical procedures, n (%)	N = 50*		
	Primary	Secondary†	Total
**Endoscopy**	28 (56)	2 (4)	30 (60)
Upper GI endoscopy with biopsy	6 (12)	0 (0)	6 (12)
Upper GI endoscopy without biopsy	15 (30)	0 (0)	15 (30)
Upper GI endoscopy with variceal banding	4 (8)	1 (2)	5 (10)
Upper GI endoscopy with variceal banding and biopsy	1 (2)	1 (2)	2 (4)
Upper GI endoscopy with variceal sclerotherapy	2 (4)	0 (0)	2 (4)
**Colonoscopy**	5 (10)	3 (6)	8 (16)
Colonoscopy with polypectomy/biopsy	3 (6)	2 (4)	5 (10)
Colonoscopy without polypectomy/biopsy	2 (4)	1 (2)	3 (6)
**Other operative procedures**	5 (10)	2 (4)	7 (14)
Primary: Two right inguinal hernia repairs, one cervical epidural injection, one right L3–L4 microdiscectomy and one endometrial curette Secondary: One umbilical hernia repair and one sigmoidoscopy			
**Liver biopsy**	4 (8)	0 (0)	4 (8)
**Dental procedure**	3 (6)	0 (0)	3 (6)
**Ablation therapy for hepatocellular carcinoma**	2 (4)	0 (0)	2 (4)
Radio-frequency ablation	2 (4)		2 (4)
**Vascular catheterization**	2 (4)	0 (0)	2 (4)
**Paracentesis**	0 (0)	1 (2)	1 (2)

Total percentages sum to > 100% as both primary and secondary procedures are listed.

GI = gastrointestinal.

* One patient received avatrombopag treatment and completed the study but did not have a procedure performed (the planned procedure was canceled).

† Secondary procedures were performed at the same time as the primary procedure.

### 3.3. Effectiveness analysis

**Figure 1.**
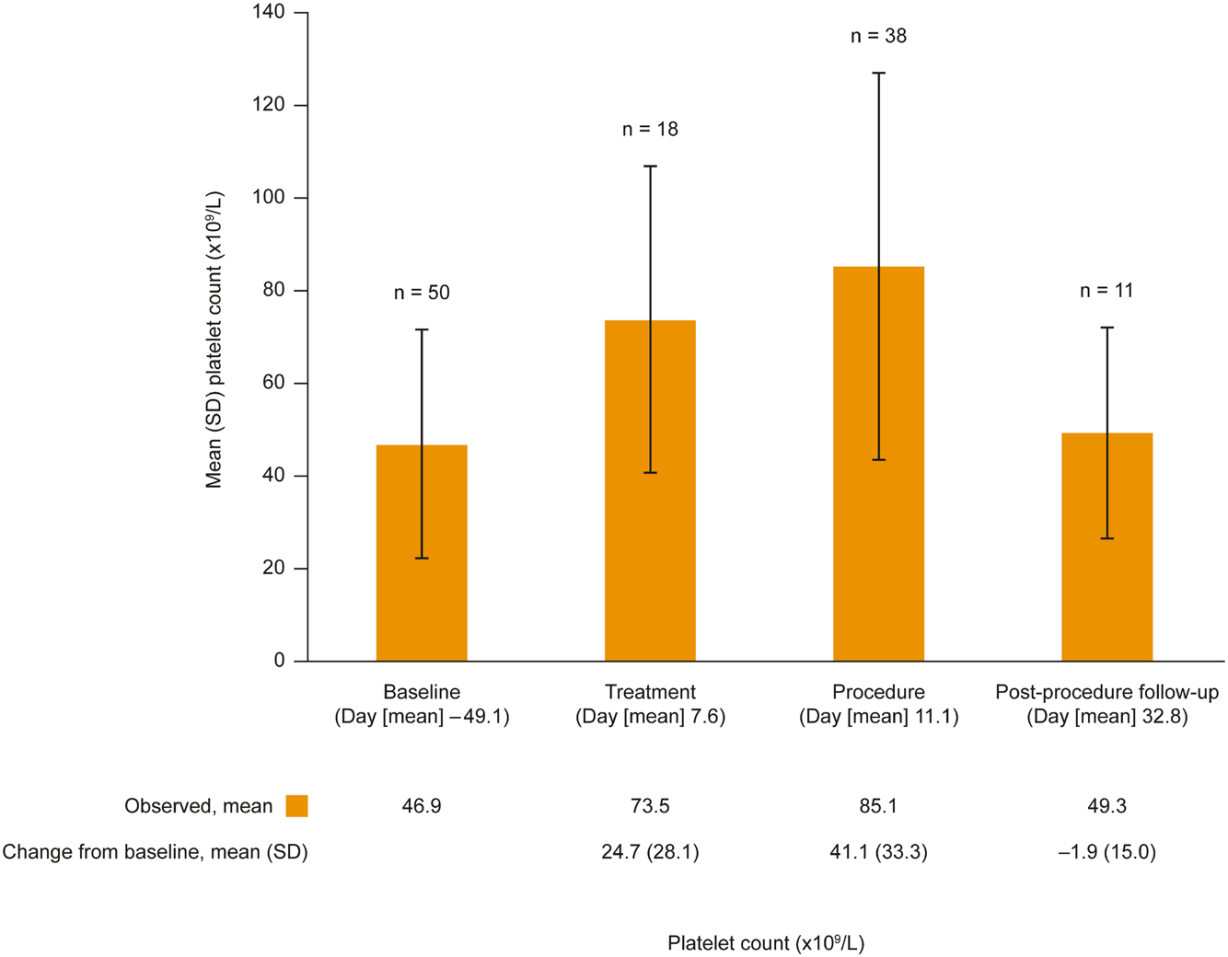
Platelet count by study visit. Treatment is defined as the day of initiation of avatrombopag up to and including the day before procedure day. For patients who did not have a platelet count assessment on the day of the procedure, but rather had a platelet count assessed the day prior to the procedure, the platelet count assessed the day prior to the procedure was summarized as a procedure platelet count. N = number, SD = standard deviation.

**Figure 2.**
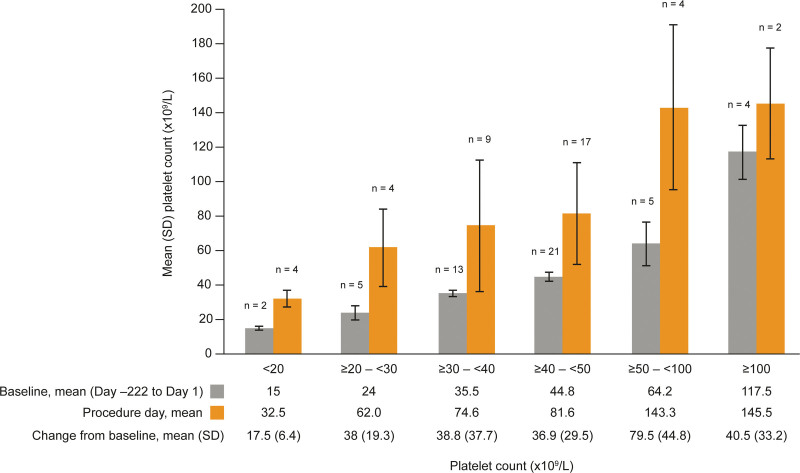
Platelet count at baseline and procedure day by baseline platelet count. For patients who did not have a platelet count assessment on the day of the procedure, but rather had a platelet count assessed the day prior to the procedure, the platelet count assessed the day prior to the procedure was summarized as a procedure platelet count. N = number, SD = standard deviation.

Treatment with avatrombopag resulted in an increased platelet count, with a mean (SD) change in platelet count from baseline to procedure day (days 8 to 15 after the first dose of avatrombopag, n = 38) of 41.1 × 10^9^/L (33.29 × 10^9^/L) (Fig. 1). The platelet count decreased upon cessation of avatrombopag treatment, with a mean (SD) change in platelet on the follow-up visit (days 11–56 after the first dose of avatrombopag, n = 11) of −1.9 × 10^9^/L (15.03 × 10^9^/L). A subgroup analysis by baseline platelet count also revealed that the mean platelet count nearly doubled, or more than doubled, from baseline to procedure day in all subgroups except in the ≥ 100 × 10^9^/L group (Fig. 2).

The proportion of patients not requiring a platelet transfusion after baseline and up to 7 calendar days following procedure day was 98% (n = 49; 95% CI: 89.4%–99.9%). One patient with a baseline platelet count of 34 × 10^9^/L received 2 units of platelets 2 days prior to the procedure (with a pre-transfusion platelet count of 46 × 10^9^/L) and 2 units of platelets on the procedure day (with a platelet count of 59 × 10^9^/L after 5 days daily treatment with 60 mg avatrombopag). This was reported as an SAE of thrombocytopenia as a result of the unplanned hospitalization to administer platelet transfusions.

### 3.4. Additional effectiveness analysis

**Figure 3.**
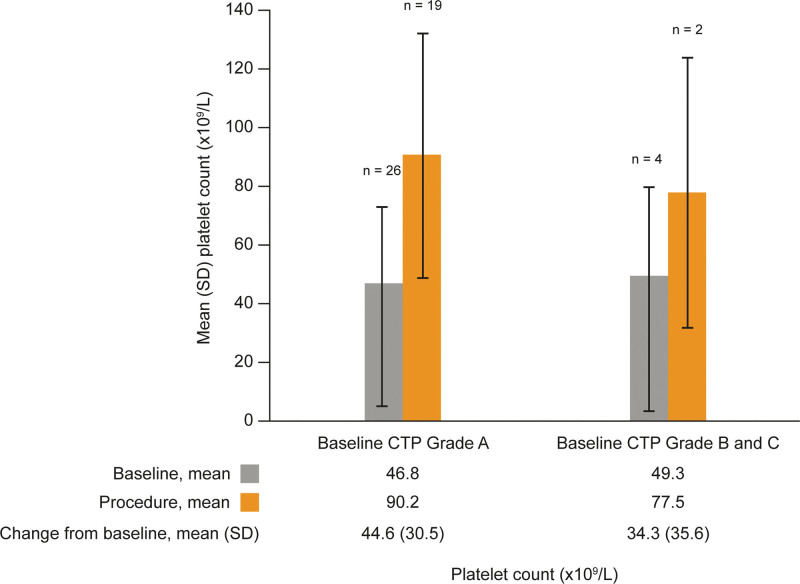
Platelet count at baseline and procedure day by Child–Turcotte–Pugh Grade. Baseline CTP Grade B and C groups were combined due to the small sample size (Grade B: baseline n = 11, procedure n = 10; Grade C: baseline n = 3, procedure n = 2). For patients who did not have a platelet count assessment on the day of the procedure, but rather had a platelet count assessed the day prior to the procedure, the platelet count assessed the day prior to the procedure was summarized as a procedure platelet count. CTP = Child–Turcotte–Pugh Grade, SD = standard deviation.

A subgroup analysis by the severity of cirrhosis, using reported CTP Grade at baseline, was performed. The change in platelet count from baseline to procedure day, within each CTP Grade, was consistent with the overall analysis (Fig. 3), although data were combined for CTP Grades B and C due to the limited numbers of patients.

### 3.5. Responder analysis

A responder analysis (patients achieving a platelet count of ≥ 50 × 10^9^/L on procedure day) was carried out for the subgroup of patients (n = 41, 82%) that had a baseline platelet count < 50 × 10^9^/L, of which 20 (40%) patients had a baseline platelet count < 40 × 10^9^/L and 21 (42%) patients had a baseline platelet count ≥ 40 to < 50 × 10^9^/L. Among these patients, 32 had a platelet count recorded on procedure day (data for the other 9 were missing) of which 27 (84.4%) achieved a platelet count ≥ 50 × 10^9^/L (Figure S1, Supplemental Digital Content, http://links.lww.com/MD/J805). In the subgroup of 35 (70%) patients that had a baseline platelet count < 50 × 10^9^/L and received a dose of avatrombopag in accordance with the US prescribing information, 18 patients had a baseline platelet count < 40 × 10^9^/L and 17 had a baseline count ≥ 40 × 10^9^/L to < 50 × 10^9^/L. Of these, 28 had a platelet count recorded on procedure day (data for the other 7 were missing) out of which 23 (82.1%) achieved a platelet count ≥ 50 × 10^9^/L.

When evaluated by baseline platelet count cohort, 9 of 18 patients with a baseline platelet count < 40 × 10^9^/L (64.3% of patients with platelet count recorded on procedure day; data for 4 were missing) and 14 of the 17 patients with a baseline platelet count ≥ 40 × 10^9^/L to < 50 × 10^9^/L (100% of patients with platelet count recorded on procedure day; data for 3 were missing) achieved a platelet count ≥ 50 × 10^9^/L on procedure day.

### 3.6. Safety analysis

All enrolled patients received 5 days of once-daily exposure to avatrombopag with no significant safety issues observed. No deaths occurred and 5 serious AEs were reported in 2 (4%) patients, with none considered related to avatrombopag treatment. The majority of patients had TEAEs that were mild (n = 3 [6%]) or moderate (n = 3 [6%]), with 1 (2%) severe TEAE of pyrexia; none led to discontinuation or were considered related to avatrombopag (Table 3). The most commonly reported TEAEs were associated with GI disorders, which was reported by 4(8%) patients (n = 4 [8%]) and the only TEAE reported in more than 1 patient was abdominal pain (n = 2 [4%]). Of the 7 (14%) patients who experienced 1 or more TEAEs, 4 (8%) patients received 60 mg and 3 (6%) patients received 40 mg daily doses of avatrombopag. Neither of the 2 patients (4%) enrolled in the study twice, who received 2 subsequent courses of avatrombopag, reported any TEAEs during the study period.

**Table 3:** Overview of treatment-emergent adverse events.

	N = 50
Number of patients with at least one TEAE, n (%)	7 (14)
Abdominal pain	2 (4)
Dyspepsia	1 (2)
Gastroesophageal reflux disease	1 (2)
Mouth hemorrhage	1 (2)
Anemia	1 (2)
Thrombocytopenia	1 (2)
Localized edema	1 (2)
Edema peripheral	1 (2)
Pyrexia	1 (2)
Arthralgia	1 (2)
Hepatic encephalopathy	1 (2)
Hemoptysis	1 (2)

TEAE = treatment-emergent adverse event.

Two mild bleeding events were reported, with both considered unrelated to avatrombopag by the investigator. One patient, prescribed 60 mg avatrombopag, had a baseline platelet count of 36 × 10^9^/L, which increased to 80 × 10^9^/L by study day 10, and had no post-procedural bleeding. On study day 47 the patient experienced hemoptysis, which was mild and resolved on the same day. Another patient, prescribed 40 mg avatrombopag, had a baseline platelet count of 47 × 10^9^/L, which increased to 167 × 10^9^/L by study day 10, and had no post-procedural bleeding. On study day 22 the patient experienced mouth hemorrhage, which was mild and resolved on the same day. No thromboembolic events were reported, and no patients received any rescue procedures (excluding transfusions) for bleeding during the study period.

## 4. Discussion

The objective of this phase 4 study was to collect real-world data on the ability of avatrombopag to increase platelet counts and reduce the need for platelet transfusions or rescue procedures for bleeding in patients with CLD scheduled to undergo a procedure. Avatrombopag was effective and well tolerated by all patients, with the mean platelet count nearly doubling by procedure day and only 1 patient (2%) requiring a platelet transfusion.

The progression of CLD frequently results in cirrhosis,^[4]^ which, as well as CLD, is associated with an increased incidence of thrombocytopenia.^[2,4]^ Any invasive procedure that is performed on a patient with CLD and thrombocytopenia carries an inherent risk of procedure-related bleeding. Historically this has been managed by platelet transfusions, but this has certain limitations.^[2,5,9]^ It is also important to note that the primary causes of thrombocytopenia in CLD are splenic platelet sequestration and breakdown, and decreased production of thrombopoietin in the liver,^[2,4]^ and therefore administering steroid treatment (the first-line treatment for immune thrombocytopenia)^[12,13]^ is inappropriate.

The advent of TPO-RAs has resulted in a new treatment management strategy to allow procedures that carry a risk of periprocedural bleeding to be managed safely in this vulnerable patient population.^[5]^ TPO-RAs have been shown to be effective at raising platelet counts, reducing the need for transfusions as well as reducing periprocedural bleeding.^[18]^ Avatrombopag is a TPO-RA that has been shown, in both the ADAPT-1 and ADAPT-2 studies, to be superior to placebo in achieving a target platelet count of 50 × 10^9^/L with a safety profile comparable to that of placebo.^[21]^

In this study, avatrombopag consistently increased platelet counts in patients with CLD and thrombocytopenia. The mean (SD) baseline platelet count in this study was 46.9 × 10^9^/L (24.52 × 10^9^/L) and the mean (SD) change in platelet count from baseline to procedure day was an increase of 41.1 × 10^9^/L (33.29 × 10^9^/L), thus demonstrating that most patients achieved the generally recommended platelet count > 50.0 × 10^9^/L on their procedure day.^[7]^ The effectiveness of avatrombopag observed in this phase 4 study in patients with a baseline platelet count < 50 × 10^9^/L is consistent with the data collected in the phase 3 ADAPT-1 (change from baseline to procedure day in platelet count of 32.0 × 10^9^/L) and ADAPT-2 (change from baseline to procedure day in platelet count of 31.3 × 10^9^/L) studies.^[21]^ Likewise, the platelet count increased following initiation of avatrombopag treatment, peaked at procedure day, and returned to near baseline levels at the post-procedure follow-up visit. Although the subgroups were small, neither the baseline platelet count nor the baseline CTP Grade appeared to impact the observed trend in platelet counts over the study period, suggesting that avatrombopag can be used to increase platelet counts prior to a scheduled procedure irrespective of the degree of thrombocytopenia or cirrhosis.

Avatrombopag was also found to reduce the need for platelet transfusions in this patient population. Forty-nine out of 50 enrolled patients did not require a platelet transfusion after the baseline visit and up to 7 days following procedure day (98%; 95% CI: 89.4%–99.9%), and no patients required a rescue procedure for bleeding during the study period. Although direct comparisons cannot be made due to the heterogeneity of patient populations and study designs, the proportion of patients not requiring a platelet transfusion was higher than that in the phase 3 ADAPT studies,^[21]^ higher than that reported for eltrombopag (72%),^[23]^ and equivalent (97.5%)^[24]^ or higher (65%–94%)^[25–28]^ than that reported for lusutrombopag.

Similar efficacy results were recently reported in a real-world retrospective study of avatrombopag in patients with CLD scheduled to undergo a procedure (n = 29), which had similar patient population demographics and in which the most common procedure was upper GI endoscopy with planned esophageal band ligation (86%).^[29]^ Patients in that study also had approximately a 2-fold increase in platelet count prior to their procedure, and no patients required rescue therapy.^[29]^

Our study provides limited initial data on the safety and effectiveness of avatrombopag in patients with baseline platelet counts greater than those previously evaluated in the phase 3 ADAPT studies. Increasing platelet counts in patients with baseline platelet counts ≥ 50 × 10^9^/L may be useful prior to more invasive surgical procedures in patients with CLD that require a higher platelet count (e.g., >100 × 10^9^/L), such as elective orthopedic surgery, craniotomy, and neurosurgery.^[10]^ In this study, 9 patients had a baseline platelet count of ≥ 50 × 10^9^/L, of which 5 had a baseline platelet count ≥ 50 × 10^9^/L and < 100 × 10^9^/L, and 4 had a baseline platelet count ≥ 100 × 10^9^/L.

The use of avatrombopag could facilitate invasive procedures that would otherwise be postponed or not performed due to the severity of the patient’s thrombocytopenia. In the real-world setting, patients with CLD require diverse interventions and severe thrombocytopenia can exclude these patients from life-saving procedures such as percutaneous radio-frequency ablation in malignant lesions.^[30]^ Several operative procedures, such as inguinal hernia repairs, that were not evaluated in the phase 3 ADAPT studies were included in this study. In this varied population of patients with CLD undergoing a range of invasive procedures, no significant bleeding events were noted, and a platelet transfusion was only required in 1 instance.

The safety profile observed in this study is comparable to the pooled phase 3 ADAPT data and similar to the reported safety of lusutrombopag.^[25,26,28]^ One of the 2 mild bleeding events was likely procedure-related and none were considered related to avatrombopag by the investigator. Importantly, no thromboembolic events were reported in any patient and no new safety signals were identified in this broader group of patients with CLD, such as patients with a platelet count ≥ 50 × 10^9^/L and patients receiving concomitant medications that were prohibited in the ADAPT studies.

The TEAEs identified in this study were similar for all doses of avatrombopag taken and do not suggest any new or unexpected safety concerns compared to the 2 large, multicenter, phase 3 ADAPT studies. The safety and effectiveness profiles did not differ in the 2 patients who received 2 subsequent courses of treatment with avatrombopag for separate procedures, in line with data published for lusutrombopag,^[28]^ and indicating that TPO-RAs are suitable for repeated use in this population.

### 4.1. Challenges and limitations

A limitation of this study is the small sample size that limits the conclusions, especially those regarding observations drawn from subgroups of patients. Nonexperimental observational studies typically involve a more diverse group of patients than experimental and interventional clinical studies and more accurately reflect real-world medical practice. Future larger real-world studies should help to elucidate the potential use of TPO-RAs prior to procedure in this challenging patient population. Further evidence may allow for shift in the risk/benefit calculation for more invasive procedures in patients with CLD-associated thrombocytopenia.

## 5. Conclusion

The results of this real-world study indicate that avatrombopag is effective in a patient population with CLD of diverse etiologies and severity. Importantly, avatrombopag was well tolerated and effective in increasing platelet counts, allowing procedures to be performed with greater confidence. The limited data presented in this study also suggest that avatrombopag is suitable for repeated use and can be used to prevent periprocedural bleeding in surgical interventions not previously studied. In a real-world setting in patients with CLD and thrombocytopenia, treatment with avatrombopag consistently increased platelet counts to nearly double the baseline platelet count by the day of the procedure and reduced the need for platelet transfusions.

## Author contributions

**Conceptualization:** Brian D Jamieson.

**Data curation:** Sanjaya K Satapathy, Vinay Sundaram, Mitchell L Shiffman, Brian D Jamieson.

**Formal analysis:** Sanjaya K Satapathy, Vinay Sundaram, Mitchell L Shiffman, Brian D Jamieson.

**Investigation:** Sanjaya K Satapathy, Vinay Sundaram, Mitchell L Shiffman, Brian D Jamieson.

**Project administration:** Sanjaya K Satapathy, Vinay Sundaram, Mitchell L Shiffman, Brian D Jamieson.

**Resources:** Sanjaya K Satapathy, Vinay Sundaram, Mitchell L Shiffman, Brian D Jamieson.

**Supervision:** Sanjaya K Satapathy, Vinay Sundaram, Mitchell L Shiffman, Brian D Jamieson.

**Validation:** Sanjaya K Satapathy, Vinay Sundaram, Mitchell L Shiffman, Brian D Jamieson.

**Writing – original draft:** Sanjaya K Satapathy, Vinay Sundaram, Mitchell L Shiffman, Brian D Jamieson.

**Writing – review & editing:** Sanjaya K Satapathy, Vinay Sundaram, Mitchell L Shiffman, Brian D Jamieson.

## Supplementary Material

## References

[1] Gkamprela E Deutsch M Pectasides D. Iron deficiency anemia in chronic liver disease: etiopathogenesis, diagnosis and treatment. Ann Gastroenterol. 2017;30:405–13. 28655976 10.20524/aog.2017.0152 PMC5479992

[2] Peck-Radosavljevic M. Thrombocytopenia in chronic liver disease. Liver Int. 2017;37:778–93. 27860293 10.1111/liv.13317

[3] Qamar AA Grace ND Groszmann RJ. Incidence, prevalence, and clinical significance of abnormal hematologic indices in compensated cirrhosis. Clin Gastroenterol Hepatol. 2009;7:689–95. 19281860 10.1016/j.cgh.2009.02.021 PMC4545534

[4] Witters P Freson K Verslype C. Review article: blood platelet number and function in chronic liver disease and cirrhosis. Aliment Pharmacol Ther. 2008;27:1017–29. 18331464 10.1111/j.1365-2036.2008.03674.x

[5] Saab S Bernstein D Hassanein T. Treatment options for thrombocytopenia in patients with chronic liver disease undergoing a scheduled procedure. J Clin Gastroenterol. 2020;54:503–11. 32195771 10.1097/MCG.0000000000001338

[6] Qureshi K Patel S Meillier A. The use of thrombopoietin receptor agonists for correction of thrombocytopenia prior to elective procedures in chronic liver diseases: review of current evidence. Int J Hepatol. 2016;2016:1802932. 27800187 10.1155/2016/1802932 PMC5075314

[7] Miller JB Figueroa EJ Haug RM. Thrombocytopenia in chronic liver disease and the role of thrombopoietin agonists. Gastroenterol Hepatol (NY). 2019;15:326–32. PMC6676354 31391802

[8] Brown RS Jr. Review article: a pharmacoeconomic analysis of thrombocytopenia in chronic liver disease. Aliment Pharmacol Ther. 2007;26(Suppl 1):41–8. 10.1111/j.1365-2036.2007.03505.x 17958518

[9] Maan R de Knegt RJ Veldt BJ. Management of thrombocytopenia in chronic liver disease: focus on pharmacotherapeutic strategies. Drugs. 2015;75:1981–92. 26501978 10.1007/s40265-015-0480-0 PMC4642582

[10] Dieterich DT Bernstein D Flamm S. Review article: a treatment algorithm for patients with chronic liver disease and severe thrombocytopenia undergoing elective medical procedures in the United States. Aliment Pharmacol Ther. 2020;52:1311–22. 32813292 10.1111/apt.16044

[11] Slichter SJ. Evidence-based platelet transfusion guidelines. Hematology Am Soc Hematol Educ Program. 2007;2007:172–8. 10.1182/asheducation-2007.1.172 18024626

[12] Provan D Arnold DM Bussel JB. Updated international consensus report on the investigation and management of primary immune thrombocytopenia. Blood Adv. 2019;3:3780–817. 31770441 10.1182/bloodadvances.2019000812 PMC6880896

[13] Neunert C Terrell DR Arnold DM. American society of hematology 2019 guidelines for immune thrombocytopenia. Blood Adv. 2019;3:3829–66. 31794604 10.1182/bloodadvances.2019000966 PMC6963252

[14] Full prescribing information for doptelet use in the treatment of thrombocytopenia in patients with chronic liver disease and in patients with chronic immune thrombocytopenia. [Dova Pharmaceuticals Web site]. Available at http://doptelet.com/themes/pdf/prescribing-information.pdf. [accessed September 27, 2021].

[15] Lusutrombopag prescribing information. Available at https://www.accessdata.fda.gov/drugsatfda_docs/label/2018/210923s000lbl.pdf. [accessed November 29, 2021].

[16] Sobi. Doptelet Summary of Product Characteristics. Available at https://www.ema.europa.eu/en/documents/product-information/doptelet-epar-product-information_en.pdf.

[17] Mulpleo summary of product characteristics. Available at https://www.ema.europa.eu/en/documents/product-information/mulpleo-epar-product-information_en.pdf. [accessed November 29, 2021.

[18] Lindquist I Olson SR Li A. The efficacy and safety of thrombopoietin receptor agonists in patients with chronic liver disease undergoing elective procedures: a systematic review and meta-analysis. Platelets. 2021;33:66–72. 33459573 10.1080/09537104.2020.1859102 PMC8286270

[19] Flisiak R Antonov K Drastich P. Practice guidelines of the central european hepatologic collaboration (CEHC) on the use of thrombopoietin receptor agonists in patients with chronic liver disease undergoing invasive procedures. J Clin Med. 2021;10:5419. 34830701 10.3390/jcm10225419 PMC8625449

[20] Rose PD Au M Woodman RJ. Pre-procedural use of thrombopoietin-receptor agonists in cirrhosis and severe thrombocytopenia: a systematic review and meta-analysis. Dig Liver Dis. 2021;53:1396–403. 34373229 10.1016/j.dld.2021.07.015

[21] Terrault N Chen YC Izumi N. Avatrombopag before procedures reduces need for platelet transfusion in patients with chronic liver disease and thrombocytopenia. Gastroenterology. 2018;155:705–18. 29778606 10.1053/j.gastro.2018.05.025

[22] Poordad F Terrault NA Alkhouri N. Avatrombopag, an alternate treatment option to reduce platelet transfusions in patients with thrombocytopenia and chronic liver disease-integrated analyses of 2 phase 3 studies. Int J Hepatol. 2020;2020:5421632. 32047671 10.1155/2020/5421632 PMC7003278

[23] Afdhal NH Giannini EG Tayyab G. Eltrombopag before procedures in patients with cirrhosis and thrombocytopenia. N Engl J Med. 2012;367:716–24. 22913681 10.1056/NEJMoa1110709

[24] Takeuchi H Furuichi Y Yoshimasu Y. The thrombopoietin receptor agonist lusutrombopag is effective for patients with chronic liver disease and impaired renal function. J Nippon Med Sch. 2021;87:325–33. 32238734 10.1272/jnms.JNMS.2020_87-603

[25] Peck-Radosavljevic M Simon K Iacobellis A. Lusutrombopag for the treatment of thrombocytopenia in patients with chronic liver disease undergoing invasive procedures (L-PLUS 2). Hepatology. 2019;70:1336–48. 30762895 10.1002/hep.30561 PMC6849531

[26] Hidaka H Kurosaki M Tanaka H. Lusutrombopag reduces need for platelet transfusion in patients with thrombocytopenia undergoing invasive procedures. Clin Gastroenterol Hepatol. 2019;17:1192–200. 30502505 10.1016/j.cgh.2018.11.047

[27] Sasaki R Shiino C Imawari M. Safety and effectiveness of lusutrombopag in Japanese chronic liver disease patients with thrombocytopenia undergoing invasive procedures: interim results of a postmarketing surveillance. Hepatol Res. 2019;49:1169–81. 31228221 10.1111/hepr.13392 PMC6899664

[28] Nishida Y Kawaoka T Imamura M. Efficacy of lusutrombopag for thrombocytopenia in patients with chronic liver disease scheduled to undergo invasive procedures. Intern Med. 2021;60:829–37. 33087674 10.2169/internalmedicine.5930-20 PMC8024946

[29] Verma D Yum JJ LeRoy K. Real-life experience with avatrombopag. Digestive Med Res. 2021;4:27.

[30] Alvaro D Caporaso N Giannini EG. Procedure-related bleeding risk in patients with cirrhosis and severe thrombocytopenia. Eur J Clin Invest. 2021;51:e13508. 33539542 10.1111/eci.13508 PMC8244048

