# Supplementary material for: Real-world use of avatrombopag in patients with chronic liver disease and thrombocytopenia undergoing a procedure
Source: Medicine (Baltimore). 2023 Oct 6;102(40):e35208. doi: 10.1097/MD.0000000000035208 (PMC10553023; doi:10.1097/MD.0000000000035208)

# Supplementary material

# Real-world use of avatrombopag in patients with chronic liver disease and thrombocytopenia undergoing a procedure

Sanjaya K. Satapathy MBBS, MD, DM, MS^a,b,^*, Vinay Sundaram MD, MSc^c^, Mitchell L. Shiffman, MD^d^, Brian D. Jamieson MD^e^

*^a^ North Shore University Hospital, Northwell Health Center for Liver Diseases & Transplantation, Northwell Health, Manhasset, NY, USA, ^b^ Department of Medicine, Donald and Barbara Zucker School of Medicine at Hofstra/Northwell Health, Hempstead, New York, USA, ^c^ Division of Gastroenterology and Comprehensive Transplant Center, Cedars-Sinai Medical Center, Los Angeles, CA, USA, ^d^ Liver Institute of Virginia, Liver Institute of Richmond, Liver Institute of Hampton Roads, Bon Secours Mercy Health, Richmond and Newport News, VA, USA, ^e^ Global Drug Development, Sobi, Durham, NC, USA*

**Supplemental Figure S1.** Patients who had a baseline platelet count of <50×10^9^/L achieving a platelet count of ≥50×10^9^/L on procedure day.


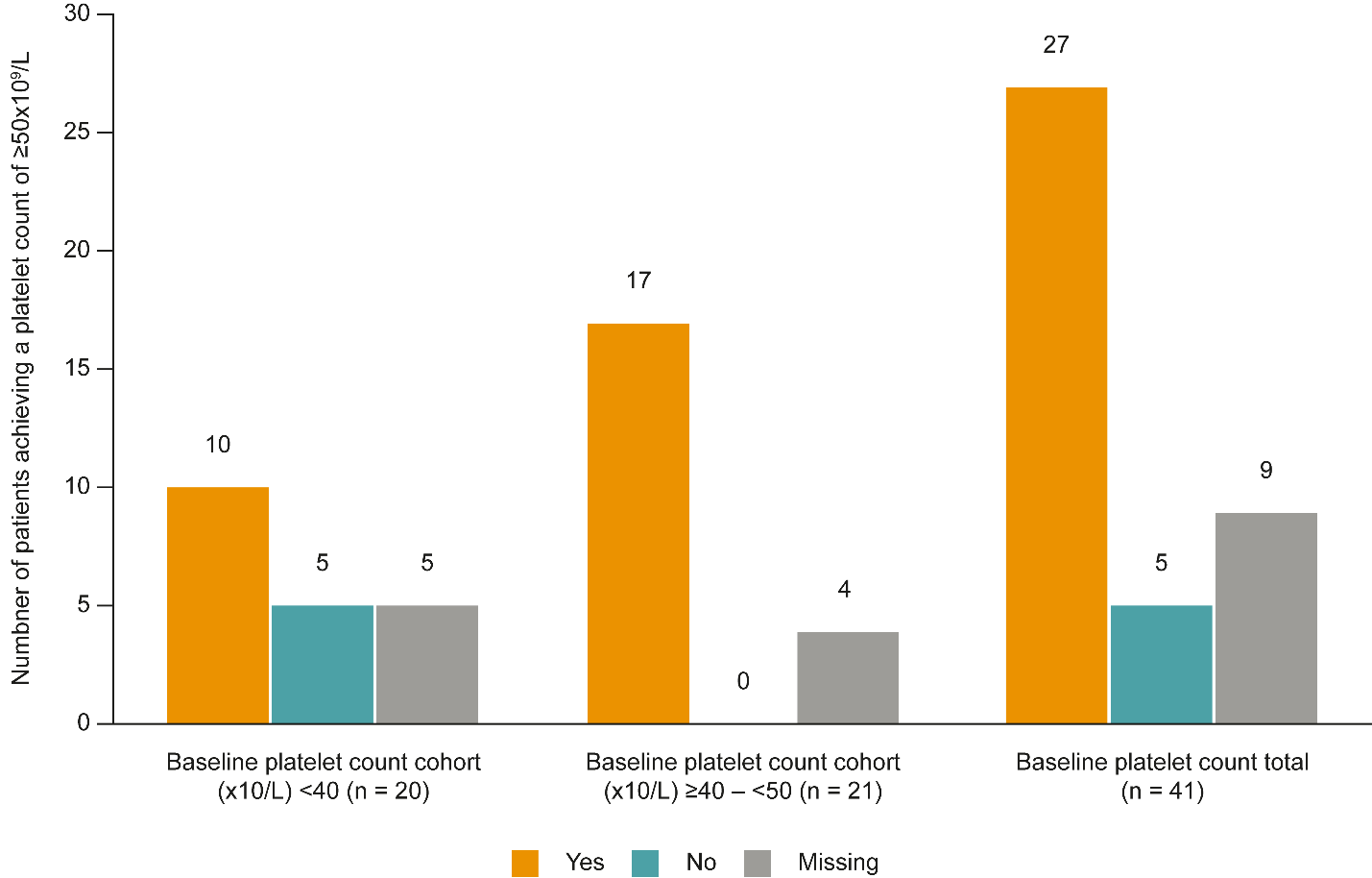

Supplement: Supplementary file 1 [file medi-102-e35208-s001.docx]
